# Supplementary material for: Improving teaching about medically unexplained symptoms for newly qualified doctors in the UK: findings from a questionnaire survey and expert workshop
Source: BMJ Open. 2017 Apr 27;7(4):e014720. doi: 10.1136/bmjopen-2016-014720 (PMC5719648; doi:10.1136/bmjopen-2016-014720)
Supplement: Supplementary data [file bmjopen-2016-014720supp001.pdf]

Thank you for taking the time to complete this short Expert Consultation Exercise. This exercise asks for your recommendations for the development of an educational intervention for FY1/FY2 doctors working with patients with medically unexplained symptoms (MUS) and should take no more than 5-10 minutes of your time.

Please note that neither you nor your institution will be identified in any publications.

We will share anonymised findings with respondents at the conclusion of the study.

Please click 'next' to begin the survey.

1. Foundation Programme:

2. NHS Trust:

3. Your Job Title (including medical speciality):

Medically unexplained symptoms (MUS) can be defined as physical symptoms where no clear organic pathology is found. Examples include symptoms such as chest pain, fatigue, palpitations and shortness of breath; and syndromes such as IBS, fibromyalgia and chronic fatigue syndrome. Sometimes other terminology is used, e.g. functional disorders.

4. Does teaching about medically unexplained symptoms (MUS) form part of the teaching that FY1/FY2 doctors receive as part of their Foundation Training Programme?

- Yes
- No

5. If so, does this take the format of a formal teaching session?

- Yes
- No
- Not Applicable

6. If yes, could you give a brief description of the content of any formal teaching given on the topic of MUS, and the amount of time spent on this?

7. Is there any reference made (either within MUS teaching or elsewhere) to the topic of 'avoiding overinvestigation'?

- Yes
- No
- Not Applicable

8. If yes, please give brief details below:

9. Do you have any formal evaluation of the teaching provided on either the topic of 'MUS' or 'avoiding overinvestigation'?

- Yes
- No
- Not Applicable

10. If yes, please give brief details of the method of evaluation used:

11. Whether or not you provide any formal teaching about 'MUS' or 'avoiding overinvestigation', do these topics come up in any case-based discussions/Balint type groups which you may run as part of your course?

- ○ Yes
- ○ No
- ○ Not Applicable

12. If so, please could you give brief details below:

13. What would you consider to be an ideal method of teaching about the topic of MUS? (Tick all that apply)

- ☐ Lecture/seminar
- ☐ Case-based group discussions
- ☐ Role play (with simulated patient)
- ☐ Role play (with peers)
- ☐ One-to-one supervision
- ☐ Ward-based teaching
- ☐ GP/outpatient based teaching
- ☐ Advanced consultation skills training

Other (please specify)

14. What would you consider to be the most feasible method of teaching about the topic of MUS? (Tick all that apply)

- [ ] Lecture/seminar
- [ ] Case-based group discussions
- [ ] Role play (with simulated patient)
- [ ] Role play (with peers)
- [ ] One-to-one supervision
- [ ] Ward-based teaching
- [ ] GP/outpatient based teaching
- [ ] Advanced consultation skills training

Other (please specify)

15. How many hours of teaching on the topic of MUS would you recommend per year at:

FY1 Level

FY2 Level

16. What barriers do you perceive to the delivery of teaching on this topic?

17. Please use the space below if you have any other comments or recommendations for teaching on this topic:

18. Would you be willing to share any details of teaching resources or evaluation methods you may have on these topics with other teachers?

- Yes
- No
- Not Applicable

19. And finally, would you be interested in taking part in a workshop to discuss the development of an educational intervention for FY1/FY2 doctors working with patients with medically unexplained symptoms (MUS)?

- Yes
- Maybe
- No

20. If you answered yes or maybe to Q18 or Q19 please provide your contact details in the space below:

Name

Email Address

Thank you very much for your recommendations
