# Supplementary material for: Improving teaching about medically unexplained symptoms for newly qualified doctors in the UK: findings from a questionnaire survey and expert workshop
Source: BMJ Open. 2017 Apr 27;7(4):e014720. doi: 10.1136/bmjopen-2016-014720 (PMC5719648; doi:10.1136/bmjopen-2016-014720)
Supplement: Author's manuscript [file bmjopen-2016-014720.draft_revisions.pdf]

BMJ Open

Medically unexplained symptoms: current teaching for newly qualified doctors in the United Kingdom and future recommendations informed by an expert workshop

Journal:	*BMJ Open*
Manuscript ID	bmjopen-2016-014720
Article Type:	Research
Date Submitted by the Author:	21-Oct-2016
Complete List of Authors:	Yon, Katherine; UCL, Research Department of Primary Care & Population Health<br>Habermann, Stephanie<br>Rosenthal, Joe; University College London Medical School,<br>Walters, Kate; University College London, Primary Care and Population Health<br>Nettleton, Sarah; University of York, Sociology<br>Warner, Alex; UCL, Research Department of Primary Care & Population Health<br>Lamahewa, Kethakie; UCL, Research Department of Primary Care & Population Health<br>Buszewicz, Marta; University College London, Research Department of Primary Care and Population Health
**Primary Subject Heading**:	Medical education and training
Secondary Subject Heading:	Communication
Keywords:	MEDICAL EDUCATION & TRAINING, MEDICALLY UNEXPLAINED SYMPTOMS, JUNIOR DOCTORS, FOUNDATION YEARS

SCHOLARONE™ Manuscripts

Medically unexplained symptoms: current teaching for newly-qualified doctors in the United Kingdom and future recommendations informed by an expert workshop

Katherine Yon[1], Stephanie Habermann[1], Joe Rosenthal[1], Kate Walters[1], Sarah Nettleton[2], Alex Warner[1], Kethakie Lamahewa[1], Marta Buszewicz[1]

[1]Research Department of Primary Care & Population Health, UCL, London, UK

[2]Department of Sociology, University of York, York, UK

Contact:
Dr Marta Buszewicz, Research Department of Primary Care and Population Health, UCL Medical School, Royal Free Campus, Upper 3rd Floor, Rowland Hill Street, London, NW3 2PF

Email: m.buszewicz@ucl.ac.uk

Tel: 020 7794 0500 (ext. 31016)

Keywords:

- Medical education & training
- Medically unexplained symptoms
- Junior doctors*
- Foundation years

*Not included in the BMJ Open list

Word Count: 3379 words (excluding title page, abstract, references, figures and tables)

For peer review only - http://bmjopen.bmj.com/site/about/guidelines.xhtml

ABSTRACT (298 words)

Objectives: Medically unexplained symptoms (MUS) present frequently in healthcare, can be complex and frustrating for clinicians and patients and are often associated with over-investigation and significant costs. Doctors need to be aware of appropriate management strategies for such patients early in their training. A previous qualitative study with Foundation year doctors (junior doctors in their first two years post-qualification) indicated significant lack of knowledge about this topic and appropriate management strategies. This study reviewed whether, and in what format, UK Foundation Training Programmes for newly-qualified doctors include any teaching about MUS and sought recommendations for further development of such training.

Design: Mixed methods design comprising a web-based questionnaire survey and an expert consultation workshop.

Setting: 19 Foundation Schools in England, Wales and Northern Ireland.

Participants: Questionnaire administered via email to 155 Foundation Training Programme Directors (FTPDs) attached to the 19 Foundation Schools, followed by an expert consultation workshop attended by 13 medical educationalists, FTPDs and junior doctors.

Results: The 53/155 (34.2%) FTPDs responding to the questionnaire represented 15 of the 19 Foundation Schools, but only 6/53 (11%) reported any current formal teaching about MUS within their programmes. However, most recognised the importance of providing such teaching, suggesting 2-3 hours per year. All those attending the expert consultation workshop recommended case-based discussions, role play and using videos to illustrate positive and negative examples of doctor-patient interactions as educational methods of choice. Educational sessions should cover the skills needed to provide appropriate explanations for patients' symptoms as well as avoiding unnecessary investigations and providing information about suitable treatment options.

For peer review only - http://bmjopen.bmj.com/site/about/guidelines.xhtml

Conclusions: There is an urgent need to improve Foundation level training about MUS, as current provision is very limited. An interactive approach covering a range of topics is recommended, but must be delivered within a realistic time frame for the curriculum.

ARTICLE SUMMARY

Strengths and limitations of this study

- To our knowledge, this is the first study to examine the provision of early postgraduate teaching on the topic of MUS for newly qualified doctors.
- This study highlights the lack of training currently taking place on this topic for UK Foundation year doctors, and draws attention to the need to include the topic in the national curriculum. This is likely to be of relevance in other countries beyond the UK.
- Only around a third of the Programme Directors approached responded to the survey, although these respondents represented 15 of the 19 Foundation Schools across England, Wales and Northern Ireland.
- Our linked studies have produced recommendations for the content and format of an educational intervention for newly-qualified doctors, but this needs to be further developed and formally evaluated to determine its impact.

For peer review only

For peer review only - http://bmjopen.bmj.com/site/about/guidelines.xhtml

INTRODUCTION

UK Medical Foundation Schools provide two years of compulsory postgraduate training for newly-qualified junior doctors. Training is delivered in accordance with the approved national curriculum developed by the UK Foundation Programme Office (UKFPO)[1], but the topics listed are fairly broad and open to local interpretation. Specifically, there is no reference to the topics of medically unexplained symptoms (MUS) or clinical uncertainty within the curriculum headings, although 'communication in difficult circumstances' is a topic likely to be of relevance to this subject.

MUS can be defined as symptoms not clearly linked to organic pathology, and include symptoms with no clear organic basis occurring within syndromes such as fibromyalgia, irritable bowel syndrome and chronic fatigue.[2] MUS are common, accounting for up to 40-50% of patients seen in primary care and up to half of patients in secondary care.[3-5] Consultations about such symptoms are often frustrating for both clinicians and patients due to the complexity and uncertainty around appropriate diagnosis and management,[6-7] and patients often express a fear of being dismissed or not believed by health professionals.[8] Unexplained symptoms are often linked to high levels of over-investigation, referrals and unnecessary treatment, placing a significant financial strain on health services,[9-10] as well as the potential for iatrogenic harm to patients.[11] It is therefore essential that doctors become aware of appropriate management strategies and treatment options available for such patients early in their careers, as they are likely to represent a significant proportion of any doctor's clinical caseload.

Recent studies have extensively reviewed the effectiveness of psychological and pharmacological treatments developed for patients with MUS,[12-13], although few studies have looked at the effectiveness of providing training for doctors working with these patients. Relevant training across undergraduate [14-15] and postgraduate [16] medical curricula within the UK is very limited, and attendance at teaching sessions is often not compulsory.

For peer review only - http://bmjopen.bmj.com/site/about/guidelines.xhtml

Training interventions for General Practitioners (GPs) within postgraduate primary care in the UK [17] and various other European countries[18-20] have been developed, but are often brief, consisting of as little as a single session. The most thoroughly evaluated GP intervention in the UK, 'the reattribution technique', focusing on reattributing physical symptoms to psycho-social causes, was found to improve doctor-patient communication but with no positive impact on patient outcomes.[21] Randomised Controlled Trials conducted in Europe examining the effects of training interventions for GPs on patient outcomes have provided mixed results, with one showing improvement in several quality of life parameters, particularly bodily pain [18], and another showing a significant reduction in patient visits at 6 month follow-up, but no improvement in patient outcomes [20].

More recently a 14 hour communication skills programme developed for qualified physicians in the Netherlands was found to improve doctor-patient communication across a variety of medical conditions, but patient outcomes were not assessed.[22] A review of recent studies looking at optimal management approaches for patients with MUS highlighted the importance of improving doctors' communication skills in this area, and emphasised the need for clinicians to understand patients' expectations in order to be able to reduce their anxiety and improve overall satisfaction.[23] Delivering effective and empowering explanations for symptoms which are meaningful to both the doctor and patient is recommended, as well as providing appropriate levels of reassurance within the context of an empathic doctor-patient relationship.[23-25]

Education about MUS should ideally begin early in a clinician's training, before management models and referral patterns are fully formed. The most opportune time may be during the years, immediately after qualification, as this is likely to be a time of significant clinical exposure to patients with MUS and junior doctors are often expected to make their own decisions regarding referrals and investigations for the first time. Effective training about the appropriate delivery of explanations for unexplained symptoms and suitable management

For peer review only - http://bmjopen.bmj.com/site/about/guidelines.xhtml

approaches is needed,[16] although there is currently little consensus as to how this training should be delivered.

This study was part of a research project funded by the National Institute for Health Research (NIHR) School of Primary Care Research (SPCR) focusing on improving training about MUS for junior doctors undertaking the UK two-year Foundation Programme (FY1/FY2). The first part involved qualitative in-depth interviews to examine junior doctors' experiences of managing patients with MUS and seek their recommendations for training and has been reported separately.[16] The second part is reported here, with the following three aims:

1. To assess to what extent teaching about MUS currently takes place within Foundation Training Programmes in England, Wales and Northern Ireland and what this involves.
2. To seek recommendations from Foundation Training Programme Directors about the content, structure and length of future teaching sessions via a questionnaire survey.
3. To hold an expert consultation workshop for professionals in order to synthesise these findings and formulate recommendations for a future educational intervention for junior doctors about managing patients with MUS.

For peer review only

For peer review only - http://bmjopen.bmj.com/site/about/guidelines.xhtml

METHODS

This study used a mixed method design incorporating a national questionnaire survey and an expert consultation workshop.

National Questionnaire Survey of Foundation Training Programme Directors (FTPDs)

Design

A web-based questionnaire entitled an 'Expert Consultation Exercise' (see Appendix) was designed comprising 14 questions. Both open and closed questions asked for information on if, when and how teaching about MUS was delivered within the various postgraduate regions, as well as any perceived barriers to delivery, and suggestions for the content, structure and length of a proposed educational intervention on this topic.

Study Population/Setting

The questionnaire was administered by email link to 155 FTPDs at Foundation Training Programmes (FTPs) across England, Wales and Northern Ireland, whose names had been obtained via FTP websites. In the majority of cases contact email addresses were not listed on the FTP websites, so email addresses were obtained via Google search.

Data Collection

Potential participants were sent an electronic request to complete the questionnaire using the online programme Survey Monkey (see http://www.surveymonkey.com). Two email reminders were sent at approximately one and two month intervals.

For peer review only

Analysis

Responses to closed questions were analysed descriptively. Responses to open questions were analysed thematically. Three members of the research team (KY, MB and SH) independently identified key themes, which were agreed by consensus.

Expert Consultation Workshop

Design

Programme Directors who had completed the questionnaire were invited to attend a three-hour expert consultation workshop, as were Foundation level trainees involved in the linked qualitative interview study on the topic [16] and also medical educationalists who had attended a previous workshop on the topic of MUS at the 2011 Association for the Study of Medical Education (ASME) conference. The workshop aimed to discuss and synthesise the findings of the national questionnaire as well as the qualitative interview data from the linked study,[16] in order to provide recommendations for the design and content of an educational intervention for junior doctors on this topic. Due to participant availability and time constraints, only one workshop date could be offered.

Workshop participants were given a summary of the questionnaire findings and the linked qualitative interview data. Participants were allocated into two groups, each with a trained facilitator (KY & MB) and encouraged to discuss the structure, content and length of future training for newly qualified doctors about MUS and any potential barriers to this. Discussion points were summarised and fed back to the main group.

Study Population/Setting

The workshop took place in Central London, UK. Thirteen people attended, including two Programme Directors, six GP educationalists, one medical sociologist, two FY2 junior doctors and two research associates representing four medical schools across the UK.

For peer review only - http://bmjopen.bmj.com/site/about/guidelines.xhtml

Data Collection

Detailed notes were taken by two members of the research team (KY & SH).

Analysis

Following the workshop, a summary of the main discussion points was collated by two members of the research team (KY & SH) and distributed amongst workshop attendees. Attendees' comments and feedback were incorporated into a final agreed summary.

RESULTS

National Questionnaire Survey

Overall, responses were received from 53/155 (34.2%) of the Foundation Training Programme Directors approached. Respondents included Programme Directors from 14 different specialties and represented 15 of the 19 Foundation Schools across England, Wales and Northern Ireland.

Current teaching on the topic of MUS

Details about current teaching taking place on the topic of MUS are documented in Table 1. Nine of the 53 Programme Directors (17%) who responded to the questionnaire indicated that they were currently providing teaching about MUS within their Foundation Schools, although this only took place as a formal teaching session in six (11%) of these programmes. Of these, two-thirds were within Greater London.

For peer review only - http://bmjopen.bmj.com/site/about/guidelines.xhtml

Table 1. Current teaching on the topic of MUS by Foundation Programmes within Foundation Schools

Question	Response
Is there teaching on MUS within FY1/2 training?	Yes (9/53) No (39/53) No response (5/53)
Is this a formal teaching session?	Yes (6/53) No (17/53) N/A (24/53) No response (10/53)
Is there any reference made to avoiding over-investigation during MUS teaching or elsewhere?	Yes (21/53) No (23/53) No response (9/53)
Do these topics arise within case-based discussion or Balint-type groups?	Yes (22/53) No (19/53) No response (12/53)

Recommendations for training

Programme Directors' recommendations regarding the proposed length and structure of an educational intervention are documented in Table 2. On average they recommended 2.2 hours during the FY1 year and 2.7 hours during the FY2 year, which would mean a dedicated teaching session on the topic each year.

Table 2. Questionnaire recommendations for future Foundation level teaching about MUS

Question	Response
What would you consider to be an ideal method of teaching? (Respondents may select more than one option)	Case-based group discussions (40/53)<br>GP/outpatient-based teaching (23/53)<br>Ward-based teaching (17/53)<br>Role play with simulated patients (14/53)<br>Advanced consultation skills training (12/53)<br>Lectures/seminars (9/53)<br>Role play with peers (8/53)<br>One-to-one supervision (6/53)

For peer review only - http://bmjopen.bmj.com/site/about/guidelines.xhtml

For peer review only

What would you consider to be the most feasible method of teaching?	Case-based group discussions (35/53)<br>Lectures (21/53)<br>GP/outpatient-based teaching (17/53)<br>Ward-based teaching (13/53)<br>Role play with peers (7/53)<br>Role play with simulated patients (6/53)<br>Advanced consultation skills training (5/53)<br>One-to-one supervision (4/53)
How many hours teaching on the topic of MUS would you recommend per year at:	FY1 level (mean: 2.24 hrs; median: 2; range: 0-10)<br>FY2 level (mean: 2.67 hrs; median: 2; range: 0-10.5)

For peer review only

Potential barriers

Most Programme Directors who responded were very much in favour of delivering teaching about MUS within the Foundation Year programme, describing this as ‘overdue’ and an important topic in need of a formal teaching commitment.

> *“[This topic] should be part of required experiential teaching for Foundation trainees.”* (P52)

A few were more cautious, and concerned that focusing on MUS might lead to junior doctors overlooking diagnoses of organic disease. The need for training to be delivered by an experienced clinician and including a focused discussion about appropriate levels of investigation was emphasised.

> *“Juniors miss straightforward physical presentations that need investigations because their heads are filled with semi-psychiatric diagnosis in lieu of the need to investigate and treat the physical problems.”* (P46)

Some potential barriers to delivering the training included issues around time constraints and timetable space, a lack of resources and appropriate facilitators, scepticism from colleagues, and junior doctor motivation to attend sessions.

For peer review only - http://bmjopen.bmj.com/site/about/guidelines.xhtml

"Time [as a barrier] - the topic requires unhurried exploration, especially including management strategies which often need to be individually 'tailored'" (P52)

However, interestingly, several Programme Directors considered that there were 'no barriers' to such training and were keen to include this topic within the postgraduate educational curriculum as soon as possible.

Expert Consultation Workshop

The topics discussed during the workshop, including recommendations for the length, content and structure of the proposed educational intervention, along with any potential barriers to its delivery are outlined below. See summary Table 3.

Table 3. Expert consultation workshop: key discussion points

Length<br>Two separate two-hour teaching sessions
Content<br>FY1: define MUS; raise awareness; emphasise clinical and economic implications<br>FY2: discuss clinical cases; provide examples of explanations for symptoms; address litigation fears and the impact of the potential negative attitudes of senior role models
Structure<br>Use of video vignettes to (i) illustrate positive and negative doctor-patient interactions and (ii) show patients' lived experience of MUS<br>Case-based group discussions<br>Role-play
Potential barriers and solutions<br>Barriers: convincing colleagues of the topic's value and time constraints within educational curricula<br>Solution: emphasise prevalence of MUS to raise awareness amongst educationalists and senior clinicians and develop the relevant educational interventions

For peer review only

Length & Content

Workshop attendees suggested the training should consist of two separate two-hour teaching sessions. The first introductory session during the first year post-qualification (FY1) should provide more factual content as a background to the topic, give definitions for the term MUS,

For peer review only - http://bmjopen.bmj.com/site/about/guidelines.xhtml

and raise awareness of both patient and clinician perspectives, with data illustrating the associated clinical and economic implications. The topic should then be revisited in the second year post-qualification (FY2), with more emphasis on specific clinical cases or issues that participating doctors had experienced in dealing with patients with unexplained symptoms, with the opportunity to discuss examples of suitable physiological and psychological explanations for common symptom presentations. Fear of litigation was considered a potential significant source of anxiety for new doctors, and the importance of addressing any such concerns emphasised.

It was also thought important to raise awareness about the different attitudes, both helpful and unhelpful, which junior doctors might encounter from their senior colleagues concerning patients with MUS, with encouragement to reflect on the potential impact of these attitudes on their own views and resulting management choices. Due to its relevance to most specialties, it was suggested that some reference to MUS should also be made wherever appropriate throughout the Foundation year educational sessions, although there was recognition that this might be difficult to implement in practice.

Structure

An innovative idea proposed by workshop attendees involved developing video vignettes to illustrate various doctor-patient interactions, e.g. positive and negative examples of role modelling when delivering explanations for common presentations of unexplained symptoms, and to show the lived experience of MUS from the patient perspective. Case-based group discussions, role-play and one-to-one supervision sessions focusing on issues around the identification and management of unexplained symptoms were also recommended. The preference was for face-to-face teaching to allow clinical case discussion, but developing an E-learning module incorporating including relevant video clips was also considered.

For peer review only - http://bmjopen.bmj.com/site/about/guidelines.xhtml

Potential barriers to overcome

Convincing colleagues involved in running local Foundation Programmes about the topic's value in an already full curriculum was identified as a significant potential barrier to providing teaching to all newly-qualified doctors. Emphasising the prevalence of MUS and raising awareness amongst educationalists and senior clinicians within relevant Trusts was identified as an important step towards its inclusion in the postgraduate curriculum.

DISCUSSION

Currently teaching about MUS is not formally listed within the curriculum for newly-qualified doctors in the UK. Very few questionnaire respondents reported any formal teaching on this topic within their Foundation Training Programmes, and less than half of respondents reported it as being informally rather than systematically discussed in case-based discussions. These findings, together with a previous survey of medical undergraduate teaching in this area, [15] and our linked study examining junior doctors' experiences of managing MUS,[16] indicate that teaching for both medical students and newly-qualified doctors about the topic of MUS is currently very limited in the UK. This highlights an urgent need to adopt a more rigorous and systematic approach to education in this area.

Most Programme Directors recognised the importance of the topic and were in favour of integrating MUS into the postgraduate training curriculum. Some were highly enthusiastic and referred to such training as long overdue, whilst a few were more cautious and concerned about the potential for junior doctors to miss cases of organic disease.

Case-based group discussions were suggested as the most favourable teaching method by both questionnaire respondents and junior doctors in our linked qualitative study.[16] Practical ward-based or outpatient-based learning was also favoured by the Programme

For peer review only - http://bmjopen.bmj.com/site/about/guidelines.xhtml

For peer review only

Directors who responded to the questionnaire, followed by role play techniques involving simulated patients or peers. These findings correspond to a comprehensive 2011 review comparing 12 systematic reviews about the teaching of communication skills to qualified physicians, which reported that the most effective programmes often involve multiple training strategies [26]. Within the review, practice-based strategies which were longer and learner-centred were seen as most effective, and interactive methods including role-play, small group discussion and feedback reported as having the most positive impact on learning. There is further evidence to suggest that interactive methods such as case-based discussions are superior to bedside teaching and lectures [27], and are a more successful method of developing knowledge, influencing workplace practice [28] and stimulating interest.[29] Although role-play exercises have received mixed reviews from learners, this has been recognised as a useful way to hone skills and practice techniques in a safe setting.[16,22]

Another teaching method suggested by workshop participants was the use of videos to illustrate various doctor-patient interactions and demonstrate both positive and negative examples of role modelling. A recent study found that, after watching videos of patients describing disease-related symptoms, medical students developed better knowledge acquisition, a deeper understanding of the problem, and showed increased interest in the patient.[30] Utilising several techniques, such as incorporating case-based discussions with video work, facilitates learning more than the use of a single technique [31-32] and can lead to improved clinical outcomes,[33] as information is reinforced through the use of different techniques which appeal to a variety of learning styles.

In the present study, workshop participants suggested providing a two-hour teaching session during the first year following qualification and then revisiting the topic during the second year, as well as referring to MUS where relevant throughout the curriculum. This is an approach supported by research showing that multiple exposure to the same subject matter

For peer review only - http://bmjopen.bmj.com/site/about/guidelines.xhtml

over time can lead to greater knowledge gain and facilitate more positive attitudes towards learning.[31-32]

In light of the results of this study and the wider literature, factors to consider for an educational intervention for newly qualified doctors include raising awareness about the topic, assisting doctors in the recognition of patients with MUS, and providing information about effective management strategies appropriate for the level of contact. This would focus mainly on the delivery of effective explanations for patients' symptoms which make sense to both the patient and the practitioner, providing appropriate reassurance and demonstrating empathy, as well as avoiding unnecessary investigations and referrals [23-25]. Highlighting that patients with MUS appear to seek emotional support more so than do other patients is also important,[6] as this may contribute to the difficulties which some of the junior doctors experienced when working with these patients. A number of these current deficiencies in training were highlighted by junior doctors in our linked qualitative study,[16] as juniors spoke about feeling stuck and unsure about how to construct and deliver suitable explanations, and feared patients' reactions to negative test results or unclear diagnoses. In light of recent Cochrane reviews examining effective psychological interventions for patients experiencing MUS (e.g. cognitive behavioural therapy or psychodynamic therapies),[12-13] raising junior doctors' awareness about possible treatments and referral options would also be an important component of training.

There is currently more evidence for the potential effectiveness of educational interventions in improving clinician skills in communicating with patients with MUS[22] and reducing health-care costs[20] than for patient health outcomes in terms of factors such as improved mood, functioning or quality of life. An educational intervention is likely to produce tangible benefits in terms of reduced frustration for both patients and clinicians, increased patient satisfaction and reduced costs for the health service, but any formal evaluation would need to assess these outcomes.

For peer review only - http://bmjopen.bmj.com/site/about/guidelines.xhtml

Studies have highlighted the impact of the negative views of some senior role models on juniors' attitudes and management choices,[14,16] drawing attention to this wider issue, and the need to bring the effective management of MUS to the attention of doctors of all levels if any training interventions are to be successfully implemented.

Strengths and limitations

To the best of our knowledge there has been no previous research into the provision of early postgraduate teaching on the topic of MUS across Foundation Schools internationally or the views of Programme Directors regarding future education in this topic. Only around a third of Programme Directors approached participated in the survey, but participants were forthcoming with both their positive views and any reservations and 15 out of 19 of the Foundation Schools across England, Wales and Northern Ireland were represented in the responses. It is possible that email addresses retrieved from the internet may be outdated, meaning a number of Programme Directors may not have received the email. As the topic of MUS does not currently feature on the core Foundation School curriculum and the actual scale of provision of teaching on the topic of MUS within the remaining Foundation Schools remains unknown, strong conclusions regarding the rates of teaching nationally cannot be confidently drawn from this data. It may be that those who responded to the questionnaire held more positive views towards MUS and its importance within the curriculum.

Implications for future research

Future research should focus on developing an educational programme aimed at newly-qualified doctors which could become part of the national curriculum, and evaluating this in terms of its impact on patient and doctor satisfaction with consultations about unexplained symptoms. It would also be important to establish whether there is a positive impact in relation to reduced cost of investigations, repeated patient attendances, and patient outcomes in terms of physical and mental health.

For peer review only - http://bmjopen.bmj.com/site/about/guidelines.xhtml

Acknowledgements

The authors would like to thank all the Foundation Training Programme Directors who took the time to complete the questionnaire, and all the participants who attended the workshop.

Competing interests

Marta Buszewicz, Kate Walters, Joe Rosenthal and Alex Warner provide lectures on the topic of MUS for fourth and fifth year medical students and Foundation year doctors in the Department of Primary Care and Population Health, UCL.

This article presents independent research funded by the National Institute for Health Research (NIHR), UK. The views expressed are those of the author(s) and not necessarily those of the NHS, the NIHR or the Department of Health. The funder has had no role in the study design, collection, analysis, and interpretation of data, writing of the manuscript or decision to submit for publication.

Funding

This work was supported by the National School of Primary Care Research (NSPCR) within the National Institute for Health Research (NIHR). Award Number: 157946.

Ethics approval

Approval from the UCL Research Ethics Committee was received on 22nd May 2014. Project ID: 4490/00.

Data Sharing Statement

An anonymised summary of the workshop discussion points and participant comments is available upon request. An anonymised summary of all of the quantitative and qualitative

For peer review only - http://bmjopen.bmj.com/site/about/guidelines.xhtml

questionnaire responses received is also available upon request from the corresponding author.

Contributors

MB, SN, KW, JR & AW developed the study protocol. All authors attended the workshop. KY collected the data. KY, MB, SN, KW, KL and SH analysed the data and interpreted the results. KY & SH drafted the report. All authors have read and approved the manuscript.

Note on contributors

Katherine Yon, BSc, MSc was a Research Associate in the Department of Primary Care and Population Health, UCL.

Stephanie Habermann, MBBS, BSc was an FY2 level junior doctor on the Royal Free Hospital training scheme at the time of the study.

Joe Rosenthal, MBBCh, MRCGP is a part-time GP and Senior Lecturer and Primary Care Education Lead in the Department of Primary Care and Population Health, UCL.

Alex Warner, MRCGP, MSc is a GP principal and Clinical Teaching Fellow in the Department of Primary Care and Population Health, UCL.

Kate Walters, MRCGP, PhD is a part-time GP and Reader in Primary Care & Epidemiology at the Department of Primary Care and Population Health, UCL.

Sarah Nettleton, BA, MSc, PhD is Professor of Sociology at the University of York, UK.

Kethakie Lamahewa was a PhD student working in the area of medically unexplained symptoms focusing on patient needs and experiences in the Department of Primary Care and Population Health, UCL.

For peer review only

Marta Buszewicz, MRCGP, MRCPsych. is a part-time GP and Reader in Primary Care at the Department of Primary Care & Population Health, UCL.

For peer review only

References

1. The Academy of Royal Medical Colleges. The UK Foundation Programme Curriculum. July 2012. http://www.foundationprogramme.nhs.uk/download.asp?file=FP_Curriculum_2012_Updated_for_Aug_2015_-_FINAL.pdf.
2. Wessley S, Nimnuan C, Sharpe M. Functional somatic syndromes: one or many? *Lancet* 1999; 354:936-9.
3. Haller H, Cramer H, Lauche R, et al. Somatoform disorders and medically unexplained symptoms in primary care: A systematic review and meta-analysis of prevalence. *Deutsches Arzteblatt International* 2015; 112:279-287.
4. Aggarwal V, Mcbeth J, Zakrzewska J, Lunt M, Macfarlane G. The epidemiology of chronic syndromes that are frequently unexplained: do they have common associated factors? *Int J Epidemiol* 2006; 35:468-476.
5. Nimnuan C, Hotopf M, Wesseley S. Medically Unexplained Symptoms: an epidemiological study in seven specialities. *J Psychosom Res* 2001; 51:361-367.
6. Salmon P, Ring A, Dowrick D, et al. What do general practice patients want when they present medically unexplained symptoms, and why do their doctors feel pressurized? *J Psychosom Res* 2005; 59:255-260.
7. Wileman L, May C, Chew-Graham CA. Medically unexplained symptoms and the problem of power in the primary care consultation: a qualitative study. *J Fam Pract* 2002; 19:178-82.
8. Peters, S, Rogers A, Salmon P, Gask L, Dowrick C, Morriss R. What do patients choose to tell their doctors? Qualitative analysis of potential barriers to reattributing medically unexplained symptoms. *J Gen Intern Med* 2009; 69:571-7.
9. Bermingham S, Cohen A, Hague J, Parsonage M. The cost of somatisation among the working-age population in England for the year 2008–2009. *Ment Health Fam Med* 2010; 7:71-84.
10. Hatcher S, Arroll B. Assessment and management of medically unexplained symptoms. *BMJ* 2008; 336:1124-1128.
11. Kouyanou, K., Pither, C. E., & Wessely, S. Iatrogenic factors and chronic pain. *Psychosomatic Medicine* 1997 59:597–604.
12. van Dessel N, den Boeft M, van der Wouden JC, Kleinstauber M, Leone SS, Terluin B, Numans ME, van der Horst HE, van Marwijk H. Non-pharmacological

For peer review only - http://bmjopen.bmj.com/site/about/guidelines.xhtml

interventions for somatoform disorders and medically unexplained physical symptoms (MUPS) in adults (Review). The Cochrane Library 2014; 11.

13. Rosendal M, Blankenstein AH, Morriss R, Fink P, Sharpe M, Burton C. Enhanced care by generalists for functional somatic symptoms and disorders in primary care (Review). The Cochrane Library 2013; 9.
14. Shattock L, Williamson H, Caldwell K, *et al.* 'They've just got symptoms without science': Medical trainees' acquisition of negative attitudes towards patients with medically unexplained symptoms. *Patient Educ Couns* 2013; 91:249-254.
15. Howman M, Walters K, Rosenthal J, *et al.* Teaching about medically unexplained symptoms at medical schools in the United Kingdom. *Med Teach* 2012; 34:327-329.
16. Yon K, Nettleton S, Walters K, Lamahewa K, Buszewicz M. Junior Doctors' Experiences of Managing Patients with Medically Unexplained Symptoms: A Qualitative Study. *BMJ Open* 2015; 5:e009593. doi:10.1136/bmjopen-2015-009593
17. Walters P, Tylee A, Fisher J, Goldberg D. Teaching junior doctors to manage patients who somatise: is it possible in an afternoon? *Med Educ* 2007; 41:995-1001.
18. Aiarzaguena JM, Grandes G, Gaminde I, Salazar A, Sanchez A, Arino J. A randomised controlled clinical trial of a psychosocial and communication intervention carried out by GPs for patients with medically unexplained symptoms. Psychological Medicine 2006; 37:283-294.
19. Rosendal M, Olesen F, Fink P, Toft T, Sokolowski I, Bro F. A randomized controlled trial of brief training in the assessment and treatment of somatization in primary care: effects on patient outcome. General Hospital Psychiatry 2007; 29:364-373.
20. Rief W, Martin A, Rauh E, Zech T, Bender A. Evaluation of general practitioners' training: how to manage patients with unexplained physical symptoms. Psychosomatics 2006; 47:304-311.
21. Morriss R, Dowrick C, Salmon P, Peters S, Dunn G, Rogers A, Lewis B, Charles-Jones H, Hogg J, Clifford R, Rigby C, Gask L. Cluster randomised controlled trial of training practices in reattribution for medically unexplained symptoms. *Brit J Psychiat* 2007; 191:536-542.
22. Weiland A, Blankenstein AH, Van Saase JLCM, Van der Molen HT, Jacobs ME, Abels DC, Kose N, Van Dulmen S, Vernhour RM, Arends LR. Training medical specialists to communicate better with patients with medically unexplained physical symptoms (MUPS). A randomized, controlled trial. *PLoS ONE* 2015; 10:e0138342. doi:10.1371/journal.pone.0138342

For peer review only - http://bmjopen.bmj.com/site/about/guidelines.xhtml

23. Weiland A, Van de Kraats RE, Blankenstein AH, Van Saase JL, Van der Molen HT, Bramer WM, Van Dulmen AM, Arends LR. Encounters between medical specialists and patients with medically unexplained physical symptoms; influences of communication on patient outcomes and use of health care: a literature overview. *Perspectives on Medical Education* 2012; 1:192-206.
24. Salmon P. Conflict, collusion of collaboration in consultations about medically unexplained symptoms: the need for a curriculum of medical explanation. *Patient Educ Couns* 2007; 67:246–54.
25. Burton C, Lucassen P, Aamland A, Olde Hartman T. Explaining symptoms after negative tests: towards a rational explanation. *J R Soc Med* 2015; 108:84–88.
26. Berkhof M, van Rijssen JH, Schellart AJM, Anema JR, van der Beek AJ. Effective training strategies for teaching communication skills to physicians: An overview of systematic reviews. *Patient Education and Counseling* 2011; 84:152-162.
27. Shahriari M. Case based teaching at the bedside versus in the classroom for undergraduates and residents of paediatrics. *J Adv Med Educ Prof* 2014; 2:135-6.
28. Bullock A, Barnes E, Ryan B, Sheen N. Case based discussion supporting learning and practice in optometry. *Ophtalmic physiol Opt* 2014; 34:614-21.
29. Onyura B, Baker L, Cameron B, Friesen F, Leslie K. Evidence for curricular and instructional design approaches in undergraduate medical education: An umbrella review. *Med Teach* 2015; 10:1–12.
30. Kommalage M, Senadheera C. Using video to introduce clinical materials. *Clin Teach* 2012; 9:248-52.
31. Bordage G, Carlin B, Mazmanian PE. Continuing medical education effect on physician knowledge: effectiveness of continuing medical education: American College of Chest Physicians Evidence-Based Educational Guidelines. *Chest* 2009; 135:29S-36S.
32. Marinopoulos SS, Dorman T, Ratanawongsa N, Wilson LM, Ashar BH, Magaziner JL, Miller RG, Thomas PA, Prokopowicz GP, Qayyum R, Bass EB. Effectiveness of continuing medical education. *Evid Rep Technol Assess* 2007; 149:1-69.
33. Mazmanian PE, Davis DA, Galbraith R. Continuing medical education effect on clinical outcomes: effectiveness of continuing medical education: American College of Chest Physicians Evidence-Based Educational Guidelines. *Chest* 2009; 135:49S-55S.

For peer review only - http://bmjopen.bmj.com/site/about/guidelines.xhtml

BMJ Open

Improving teaching about medically unexplained symptoms for newly-qualified doctors in the UK: findings from a questionnaire survey and expert workshop

Journal:	*BMJ Open*
Manuscript ID	bmjopen-2016-014720.R1
Article Type:	Research
Date Submitted by the Author:	13-Feb-2017
Complete List of Authors:	Yon, Katherine; UCL, Research Department of Primary Care & Population Health<br>Habermann, Stephanie<br>Rosenthal, Joe; University College London Medical School,<br>Walters, Kate; University College London, Primary Care and Population Health<br>Nettleton, Sarah; University of York, Sociology<br>Warner, Alex; UCL, Research Department of Primary Care & Population Health<br>Lamahewa, Kethakie; UCL, Research Department of Primary Care & Population Health<br>Buszewicz, Marta; University College London, Research Department of Primary Care and Population Health
**Primary Subject Heading**:	Medical education and training
Secondary Subject Heading:	Communication
Keywords:	MEDICAL EDUCATION & TRAINING, MEDICALLY UNEXPLAINED SYMPTOMS, JUNIOR DOCTORS, FOUNDATION YEARS

SCHOLARONE™
Manuscripts

Improving teaching about medically unexplained symptoms for newly-qualified doctors in the UK: findings from a questionnaire survey and expert workshop

Katherine Yon[1], Stephanie Habermann[1], Joe Rosenthal[1], Kate Walters[1], Sarah Nettleton[2], Alex Warner[1], Kethakie Lamahewa[1], Marta Buszewicz[1]

[1]Research Department of Primary Care & Population Health, UCL, London, UK

[2]Department of Sociology, University of York, York, UK

Contact:

Dr Marta Buszewicz, Research Department of Primary Care and Population Health, UCL Medical School, Royal Free Campus, Upper 3rd Floor, Rowland Hill Street, London, NW3 2PF

Email: m.buszewicz@ucl.ac.uk

Tel: 020 7794 0500 (ext. 31016)

Keywords:

- Medical education & training
- Medically unexplained symptoms
- Junior doctors*
- Foundation years

*Not included in the BMJ Open list

Word Count: 3367 words (excluding title page, abstract, references, figures and tables)

For peer review only

ABSTRACT (298 words)

Objectives: Medically unexplained symptoms (MUS) present frequently in healthcare, can be complex and frustrating for clinicians and patients and are often associated with over-investigation and significant costs. Doctors need to be aware of appropriate management strategies for such patients early in their training. A previous qualitative study with Foundation year doctors (junior doctors in their first two years post-qualification) indicated significant lack of knowledge about this topic and appropriate management strategies. This study reviewed whether, and in what format, UK Foundation Training Programmes for newly-qualified doctors include any teaching about MUS and sought recommendations for further development of such training.

Design: Mixed methods design comprising a web-based questionnaire survey and an expert consultation workshop.

Setting: 19 Foundation Schools in England, Wales and Northern Ireland.

Participants: Questionnaire administered via email to 155 Foundation Training Programme Directors (FTPDs) attached to the 19 Foundation Schools, followed by an expert consultation workshop attended by 13 medical educationalists, FTPDs and junior doctors.

Results: The 53/155 (34.2%) FTPDs responding to the questionnaire represented 15 of the 19 Foundation Schools, but only 6/53 (11%) reported any current formal teaching about MUS within their programmes. However, most recognised the importance of providing such teaching, suggesting 2-3 hours per year. All those attending the expert consultation workshop recommended case-based discussions, role play and using videos to illustrate positive and negative examples of doctor-patient interactions as educational methods of choice. Educational sessions should cover the skills needed to provide appropriate explanations for patients' symptoms as well as avoiding unnecessary investigations and providing information about suitable treatment options.

For peer review only - http://bmjopen.bmj.com/site/about/guidelines.xhtml

Conclusions: There is an urgent need to improve Foundation level training about MUS, as current provision is very limited. An interactive approach covering a range of topics is recommended, but must be delivered within a realistic time frame for the curriculum.

ARTICLE SUMMARY

Strengths and limitations of this study

- To our knowledge, this is the first study to examine the provision of early postgraduate teaching on the topic of MUS for newly qualified doctors.
- This study highlights the lack of training currently taking place on this topic for UK Foundation year doctors, and draws attention to the need to include the topic in the national curriculum. This is likely to be of relevance in other countries beyond the UK.
- Only around a third of the Programme Directors approached responded to the survey, although these respondents represented 15 of the 19 Foundation Schools across England, Wales and Northern Ireland.
- Our linked studies have produced recommendations for the content and format of an educational intervention for newly-qualified doctors, but this needs to be further developed and formally evaluated to determine its impact.

For peer review only - http://bmjopen.bmj.com/site/about/guidelines.xhtml

INTRODUCTION

UK Medical Foundation Schools provide two years of compulsory postgraduate training for newly-qualified junior doctors. Training is delivered in accordance with the approved national curriculum developed by the UK Foundation Programme Office (UKFPO)[1], but the topics listed are fairly broad and open to local interpretation. Specifically, there is no reference to the topics of medically unexplained symptoms (MUS) or clinical uncertainty within the curriculum headings, although 'communication in difficult circumstances' is likely to be of relevance to this subject.

MUS can be defined as symptoms not clearly linked to organic pathology, and include symptoms with no clear organic basis occurring within syndromes such as fibromyalgia, irritable bowel syndrome and chronic fatigue.[2] MUS are common, accounting for up to 40-50% of patients seen in primary care and up to half of patients in secondary care.[3-5] Consultations about such symptoms are often frustrating for both clinicians and patients due to the complexity and uncertainty around appropriate diagnosis and management,[6-7] and patients often express a fear of being dismissed or not believed by health professionals.[8] Unexplained symptoms are often linked to high levels of over-investigation, referrals and unnecessary treatment, placing a significant financial strain on health services,[9-10] as well as the potential for iatrogenic harm to patients.[11] It is therefore essential that doctors become aware of appropriate management strategies and treatment options available for such patients early in their careers, as they are likely to represent a significant proportion of any doctor's clinical caseload.

Recent studies have extensively reviewed the effectiveness of psychological and pharmacological treatments developed for patients with MUS,[12-13], although few studies have looked at the effectiveness of providing training for doctors working with these patients. Relevant training across undergraduate [14-15] and postgraduate [16] medical curricula within the UK is very limited, and attendance at teaching sessions is often not compulsory.

For peer review only - http://bmjopen.bmj.com/site/about/guidelines.xhtml

Training interventions for General Practitioners (GPs) within postgraduate primary care in the UK [17] and various other European countries[18-20] have been developed, but are often brief, consisting of as little as a single session. The most thoroughly evaluated GP intervention in the UK, 'the reattribution technique', focusing on reattributing physical symptoms to psycho-social causes, was found to improve doctor-patient communication but with no positive impact on patient outcomes.[21] Randomised Controlled Trials conducted in Europe examining the effects of training interventions for GPs on patient outcomes have provided mixed results, with one showing improvement in several quality of life parameters, particularly bodily pain [18], and another showing a significant reduction in patient visits at 6 month follow-up, but no improvement in patient outcomes [20].

More recently a 14 hour communication skills programme developed for qualified physicians in the Netherlands was found to improve doctor-patient communication across a variety of medical conditions, but patient outcomes were not assessed.[22] A review of recent studies looking at optimal management approaches for patients with MUS highlighted the importance of improving doctors' communication skills in this area, and emphasised the need for clinicians to understand patients' expectations in order to be able to reduce their anxiety and improve overall satisfaction.[23] Delivering effective and empowering explanations for symptoms which are meaningful to both the doctor and patient is recommended, as well as providing appropriate levels of reassurance within the context of an empathic doctor-patient relationship.[23-25]

Education about MUS should ideally begin early in a clinician's training, before management models and referral patterns are fully formed. The most opportune time may be during the early years immediately after qualification, as this is likely to be a time of significant clinical exposure to patients with MUS and junior doctors are often expected to make their own decisions regarding referrals and investigations for the first time. Effective training about the appropriate delivery of explanations for unexplained symptoms and suitable management

For peer review only - http://bmjopen.bmj.com/site/about/guidelines.xhtml

approaches is needed,[16] although there is currently little consensus as to how this training should be delivered.

This study was part of a research project funded by the National Institute for Health Research (NIHR) School of Primary Care Research (SPCR) focusing on improving training about MUS for junior doctors undertaking the UK two-year Foundation Programme (FY1/FY2). The first part involved qualitative in-depth interviews to examine junior doctors' experiences of managing patients with MUS and seek their recommendations for training and has been reported separately.[16] The second part is reported here, with the following three aims:

1. To assess to what extent teaching about MUS currently takes place within Foundation Training Programmes in England, Wales and Northern Ireland and what this involves.
2. To seek recommendations from Foundation Training Programme Directors about the content, structure and length of future teaching sessions via a questionnaire survey.
3. To hold an expert consultation workshop for professionals in order to synthesise these findings and formulate recommendations for a future educational intervention for junior doctors about managing patients with MUS.

For peer review only

For peer review only - http://bmjopen.bmj.com/site/about/guidelines.xhtml

METHODS

This study used a mixed method design incorporating a national questionnaire survey and an expert consultation workshop.

National Questionnaire Survey of Foundation Training Programme Directors (FTPDs)

Design

A web-based questionnaire entitled an 'Expert Consultation Exercise' (see Appendix) was designed comprising 14 questions. Its content was informed by the findings of the linked qualitative study [16] and an earlier survey of the provision of undergraduate teaching about MUS in UK Medical Schools [15]. Both open and closed questions asked for information on if, when and how teaching about MUS was delivered within the various postgraduate regions, as well as any perceived barriers to delivery, and suggestions for the content, structure and length of a proposed educational intervention on this topic.

Study Population/Setting

The questionnaire was administered by email link to 155 FTPDs at Foundation Training Programmes (FTPs) across England, Wales and Northern Ireland, whose names had been obtained via FTP websites. In the majority of cases contact email addresses were not listed on the FTP websites, so email addresses were obtained via Google search.

Data Collection

Potential participants were sent an electronic request to complete the questionnaire using the online programme Survey Monkey (see http://www.surveymonkey.com). Two email reminders were sent at approximately one and two month intervals.

For peer review only - http://bmjopen.bmj.com/site/about/guidelines.xhtml

Analysis

Responses to closed questions have been presented as descriptive statistics. Responses to open questions were analysed thematically in order to identify key themes emerging from the data. Three members of the research team (KY, MB and SH) independently identified key themes emerging from the dataset, then met and defined themes by consensus.

Expert Consultation Workshop

Design

Programme Directors who had completed the questionnaire were invited to attend a three-hour expert consultation workshop, as were Foundation level trainees involved in the linked qualitative interview study on the topic [16] and also medical educationalists who had attended a previous workshop on the topic of MUS at the 2011 Association for the Study of Medical Education (ASME) conference. The expert workshop aimed to discuss and synthesise the findings of the national questionnaire and the qualitative interview data from the linked study,[16] in order to provide recommendations for the design and content of an educational intervention for junior doctors on this topic. Due to participant availability and time constraints, only one workshop date could be offered.

Workshop participants were given a summary of the questionnaire findings and the linked qualitative interview data. Participants were allocated into two groups, each with a trained facilitator (KY & MB) and encouraged to discuss the structure, content and length of future training for newly qualified doctors about MUS and any potential barriers to this. Discussion points were summarised and fed back to the main group.

For peer review only - http://bmjopen.bmj.com/site/about/guidelines.xhtml

Study Population/Setting

The workshop took place in Central London, UK. Thirteen people attended, including two Programme Directors, six GP educationalists, one medical sociologist, two FY2 junior doctors and two research associates representing four medical schools across the UK.

Data Collection

Detailed notes were taken by two members of the research team (KY & SH).

Analysis

Following the workshop, a summary of the main discussion points was collated by two members of the research team (KY & SH) and distributed amongst workshop attendees. Attendees' comments and feedback were incorporated into a final agreed summary.

RESULTS

National Questionnaire Survey

Overall, responses were received from 53/155 (34.2%) of the Foundation Training Programme Directors approached. Respondents included Programme Directors from 14 different specialties and represented 15 of the 19 Foundation Schools across England, Wales and Northern Ireland.

Current teaching on the topic of MUS

Details about current teaching taking place are documented in Table 1. Nine of the 53 Programme Directors (17%) who responded to the questionnaire indicated that they were currently providing teaching about MUS within their Foundation Schools, although this only took place as a formal teaching session in six (11%) of these programmes. Of these, two-thirds were within Greater London.

For peer review only

Table 1. Current teaching on the topic of MUS by Foundation Programmes within Foundation Schools

Question	Response
Is there teaching on MUS within FY1/2 training?	Yes (9/53) No (39/53) No response (5/53)
Is this a formal teaching session?	Yes (6/53) No (17/53) N/A (24/53) No response (10/53)
Is there any reference made to avoiding over-investigation during MUS teaching or elsewhere?	Yes (21/53) No (23/53) No response (9/53)
Do these topics arise within case-based discussion or Balint-type groups?	Yes (22/53) No (19/53) No response (12/53)

Recommendations for training

Programme Directors' recommendations regarding the proposed length and structure of an educational intervention are documented in Table 2. On average they recommended 2.2 hours during the FY1 year and 2.7 hours during the FY2 year, which would mean a dedicated teaching session on the topic each year.

For peer review only - http://bmjopen.bmj.com/site/about/guidelines.xhtml

Table 2. Questionnaire recommendations for future Foundation level teaching about MUS

Question	Response
What would you consider to be an ideal method of teaching? (Respondents may select more than one option)	Case-based group discussions (40/53)<br>GP/outpatient-based teaching (23/53)<br>Ward-based teaching (17/53)<br>Role play with simulated patients (14/53)<br>Advanced consultation skills training (12/53)<br>Lectures/seminars (9/53)<br>Role play with peers (8/53)<br>One-to-one supervision (6/53)
What would you consider to be the most feasible method of teaching?	Case-based group discussions (35/53)<br>Lectures (21/53)<br>GP/outpatient-based teaching (17/53)<br>Ward-based teaching (13/53)<br>Role play with peers (7/53)<br>Role play with simulated patients (6/53)<br>Advanced consultation skills training (5/53)<br>One-to-one supervision (4/53)
How many hours teaching on the topic of MUS would you recommend per year at:	FY1 level (mean: 2.24 hrs; median: 2; range: 0-10)<br>FY2 level (mean: 2.67 hrs; median: 2; range: 0-10.5)

Potential barriers

Most Programme Directors who responded were very much in favour of delivering teaching about MUS within the Foundation Year programme, describing this as 'overdue' and an important topic in need of a formal teaching commitment.

> *"[This topic] should be part of required experiential teaching for Foundation trainees."* (P52)

A few were more cautious, and concerned that focusing on MUS might lead to junior doctors overlooking diagnoses of organic disease. The need for training to be delivered by an experienced clinician and including a focused discussion about appropriate levels of investigation was emphasised.

For peer review only - http://bmjopen.bmj.com/site/about/guidelines.xhtml

"Juniors miss straightforward physical presentations that need investigations because their heads are filled with semi-psychiatric diagnosis in lieu of the need to investigate and treat the physical problems." (P46)

Some potential barriers to delivering the training included issues around time constraints and timetable space, a lack of resources and appropriate facilitators, scepticism from colleagues, and junior doctor motivation to attend sessions.

"Time [as a barrier] - the topic requires unhurried exploration, especially including management strategies which often need to be individually 'tailored'" (P52)

However, interestingly, several Programme Directors considered that there were 'no barriers' to such training and were keen to include this topic within the postgraduate educational curriculum as soon as possible.

Expert Consultation Workshop

The topics discussed during the workshop, including recommendations for the length, content and structure of the proposed educational intervention, along with potential barriers to its delivery are outlined below in Table 3.

For peer review only

Table 3. Expert consultation workshop: key discussion points

Length
Two separate two-hour teaching sessions

Content
FY1: define MUS; raise awareness; emphasise clinical and economic implications
FY2: discuss clinical cases; provide examples of explanations for symptoms; address litigation fears and the impact of the potential negative attitudes of senior role models

Structure
Use of video vignettes to (i) illustrate positive and negative doctor-patient interactions and (ii) show patients' lived experience of MUS
Case-based group discussions
Role-play

Potential barriers and solutions
Barriers: convincing colleagues of the topic's value and time constraints within educational curricula
Solution: emphasise prevalence of MUS to raise awareness amongst educationalists and senior clinicians and develop the relevant educational interventions

Length & Content

Workshop attendees suggested the training should consist of two separate two-hour teaching sessions. The first introductory session during the first year post-qualification (FY1) should provide more factual content as a background to the topic, give definitions for the term MUS, and raise awareness of both patient and clinician perspectives, with data illustrating the associated clinical and economic implications. The topic should then be revisited in the second year post-qualification (FY2), with more emphasis on specific clinical cases or issues that participating doctors had experienced in dealing with patients with unexplained symptoms, with the opportunity to discuss examples of suitable physiological and psychological explanations for common symptom presentations. Fear of litigation was considered a potential significant source of anxiety for new doctors, and the importance of addressing any such concerns emphasised.

For peer review only - http://bmjopen.bmj.com/site/about/guidelines.xhtml

It was also thought important to raise awareness about the different attitudes, both helpful and unhelpful, which junior doctors might encounter from their senior colleagues concerning patients with MUS, with encouragement to reflect on the potential impact of these attitudes on their own views and resulting management choices. Due to its relevance to most specialties, it was suggested that some reference to MUS should also be made wherever appropriate throughout Foundation year educational sessions, although it was recognised this might be difficult to implement in practice.

Structure

An innovative idea proposed by workshop attendees involved developing video vignettes to illustrate various doctor-patient interactions, e.g. positive and negative examples of role modelling when delivering explanations for common presentations of unexplained symptoms, and to show the lived experience of MUS from the patient perspective. Case-based group discussions, role-play and one-to-one supervision sessions focusing on issues around the identification and management of unexplained symptoms were also recommended. The preference was for face-to-face teaching to allow clinical case discussion, but developing an E-learning module incorporating including relevant video clips was also considered.

Potential barriers to overcome

Convincing colleagues involved in running local Foundation Programmes about the topic's value in an already full curriculum was identified as a significant potential barrier to providing teaching to all newly-qualified doctors. Emphasising the prevalence of MUS and raising awareness amongst educationalists and senior clinicians within relevant Trusts was identified as an important step towards its inclusion in the postgraduate curriculum.

For peer review only - http://bmjopen.bmj.com/site/about/guidelines.xhtml

DISCUSSION

Currently teaching about MUS is not formally listed within the curriculum for newly-qualified doctors in the UK. Very few questionnaire respondents reported any formal teaching on this topic within their Foundation Training Programmes, and less than half of respondents reported it as being informally rather than systematically discussed in case-based discussions. These findings, together with a previous survey of medical undergraduate teaching in this area, [15] and our linked study examining junior doctors' experiences of managing MUS,[16] indicate that teaching for both medical students and newly-qualified doctors about this topic is currently very limited in the UK. This highlights an urgent need to adopt a more rigorous and systematic approach to education in this area.

Most Programme Directors recognised the importance of the topic and were in favour of integrating MUS into the postgraduate training curriculum. Some were highly enthusiastic and referred to such training as long overdue, whilst a few were more cautious and concerned about the potential for junior doctors to miss cases of organic disease.

Case-based group discussions were recommended as the most favourable teaching method by both questionnaire respondents and junior doctors in our linked qualitative study.[16] Practical ward-based or outpatient-based learning was also favoured by the Programme Directors who responded to the questionnaire, followed by role play techniques involving simulated patients or peers. These findings correspond to a comprehensive 2011 review comparing 12 systematic reviews about the teaching of communication skills to qualified physicians, which reported that the most effective programmes often involve multiple training strategies.[26] Within the review, practice-based strategies which were longer and learner-centred were seen as most effective, and interactive methods including role-play, small group discussion and feedback reported as having the most positive impact on learning. There is further evidence to suggest interactive methods such as case-based discussions are

For peer review only - http://bmjopen.bmj.com/site/about/guidelines.xhtml

superior to bedside teaching and lectures,[27] and a more successful method of developing knowledge, influencing workplace practice[28] and stimulating interest.[29] Although role-play exercises have received mixed reviews from learners, this has been recognised as a useful way to hone skills and practice techniques in a safe setting.[16,22]

Another teaching method suggested by workshop participants was the use of videos to illustrate various doctor-patient interactions and demonstrate both positive and negative examples of role modelling. A recent study found that, after watching videos of patients describing disease-related symptoms, medical students developed better knowledge acquisition, a deeper understanding of the problem, and showed increased interest in the patient.[30] Utilising several techniques, such as incorporating case-based discussions with video work, facilitates learning more than the use of a single technique [31-32] and can lead to improved clinical outcomes,[33] as information is reinforced through the use of different techniques which appeal to a variety of learning styles.

In the present study, workshop participants suggested providing a two-hour teaching session during the first year following qualification and then revisiting the topic during the second year, as well as referring to MUS where relevant throughout the curriculum. This is an approach supported by research showing that multiple exposure to the same subject matter over time can lead to greater knowledge gain and facilitate more positive attitudes towards learning.[31-32]

In light of the results of this study and the wider literature, factors to consider for an educational intervention for newly qualified doctors include raising awareness about the topic, assisting doctors in the recognition of patients with MUS, and providing information about effective management strategies appropriate for the level of contact. This would focus mainly on accurately identifying the problem and the patient's concerns, giving effective explanations for symptoms which make sense to both the patient and the practitioner,

For peer review only - http://bmjopen.bmj.com/site/about/guidelines.xhtml

providing appropriate reassurance and demonstrating empathy, as well as avoiding unnecessary investigations and referrals.[23-25] Highlighting that patients with MUS appear to seek emotional support more than other patients is also important,[6] as this may contribute to the difficulties some of the junior doctors experienced when working with these patients. A number of these current deficiencies in training were highlighted by junior doctors in our linked qualitative study[16] as juniors spoke about feeling stuck and unsure about how to construct and deliver suitable explanations, and feared patients' reactions to negative test results or unclear diagnoses. Including teaching about various explanatory models of MUS, such as somatosensory amplification theory, immune system sensitisation theory and various cognitive theories [34], could also be useful to encourage doctors to think about providing explanations for MUS within a biopsychosocial context [16]. In light of recent Cochrane reviews examining effective psychological interventions for patients experiencing MUS (e.g. cognitive behavioural therapy or psychodynamic therapies),[12-13] raising junior doctors' awareness about possible treatments and referral options would be an important component of training.

It is important to highlight that, although specific management techniques have been recommended in our paper and in the literature, there is currently only clear evidence for their effectiveness in improving clinician skills when communicating with patients with MUS[22] and reducing investigations and health-care costs[20]. An educational intervention focusing on these areas is likely to produce tangible benefits in terms of reduced frustration for both patients and clinicians, increased patient satisfaction and reduced costs. The evidence for a direct impact on clinical outcomes such as improved mood, functioning or quality of life is still lacking and any formal evaluation of a new educational intervention would need to carefully assess these factors. The combination of videos and case-based learning which we are suggesting is a novel approach and might prove more effective at impacting on clinical outcomes given the evidence for such a combined approach.[31-33]

For peer review only - http://bmjopen.bmj.com/site/about/guidelines.xhtml

For peer review only

Studies have highlighted the impact of the negative views of some senior role models on juniors' attitudes and management choices,[14,16] drawing attention to this wider issue, and the need to bring the effective management of MUS to the attention of doctors of all levels if any training interventions are to be successfully implemented.

Strengths and limitations

To the best of our knowledge there has been no previous research into the provision of early postgraduate teaching on the topic of MUS across Foundation Schools internationally or the views of Programme Directors regarding future education in this topic. Only around a third of Programme Directors approached participated in the survey, but participants were forthcoming with both their positive views and any reservations and 15 out of 19 of the Foundation Schools across England, Wales and Northern Ireland were represented in the responses. It is possible that email addresses retrieved from the internet may be outdated, meaning a number of Programme Directors may not have received the email. As the topic of MUS does not currently feature on the core Foundation School curriculum and the actual scale of provision of teaching on the topic of MUS within the remaining Foundation Schools remains unknown, strong conclusions regarding the rates of teaching nationally cannot be confidently drawn from this data. It may be that those who responded to the questionnaire held more positive views towards MUS and its importance within the curriculum.

Implications for future research

Future research should focus on developing an educational programme aimed at newly-qualified doctors which could become part of the national curriculum, and evaluating this in terms of its impact on patient and doctor satisfaction with consultations about unexplained symptoms. It would also be important to establish whether there is a positive impact in relation to reduced cost of investigations, repeated patient attendances, and patient outcomes in terms of physical and mental health.

For peer review only - http://bmjopen.bmj.com/site/about/guidelines.xhtml

Acknowledgements

The authors would like to thank all the Foundation Training Programme Directors who took the time to complete the questionnaire, and all the participants who attended the workshop.

Competing interests

Marta Buszewicz, Kate Walters, Joe Rosenthal and Alex Warner provide lectures on the topic of MUS for fourth and fifth year medical students and Foundation year doctors in the Department of Primary Care and Population Health, UCL.

This article presents independent research funded by the National Institute for Health Research (NIHR), UK. The views expressed are those of the author(s) and not necessarily those of the NHS, the NIHR or the Department of Health. The funder has had no role in the study design, collection, analysis, and interpretation of data, writing of the manuscript or decision to submit for publication.

Funding

This work was supported by the National School of Primary Care Research (NSPCR) within the National Institute for Health Research (NIHR). Award Number: 157946.

Ethics approval

Approval from the UCL Research Ethics Committee was received on 22nd May 2014. Project ID: 4490/00.

Data Sharing Statement

An anonymised summary of the workshop discussion points and participant comments is available upon request. An anonymised summary of all of the quantitative and qualitative

For peer review only - http://bmjopen.bmj.com/site/about/guidelines.xhtml

questionnaire responses received is also available upon request from the corresponding author.

Contributors

MB, SN, KW, JR & AW developed the study protocol. All authors attended the workshop. KY collected the data. KY, MB, SN, KW, KL and SH analysed the data and interpreted the results. KY & SH drafted the report. All authors have read and approved the manuscript.

Note on contributors

Katherine Yon, BSc, MSc was a Research Associate in the Department of Primary Care and Population Health, UCL.

Stephanie Habermann, MBBS, BSc was an FY2 level junior doctor on the Royal Free Hospital training scheme at the time of the study.

Joe Rosenthal, MBBCh, MRCGP is a part-time GP and Senior Lecturer and Primary Care Education Lead in the Department of Primary Care and Population Health, UCL.

Alex Warner, MRCGP, MSc is a GP principal and Clinical Teaching Fellow in the Department of Primary Care and Population Health, UCL.

Kate Walters, MRCGP, PhD is a part-time GP and Reader in Primary Care & Epidemiology at the Department of Primary Care and Population Health, UCL.

Sarah Nettleton, BA, MSc, PhD is Professor of Sociology at the University of York, UK.

Kethakie Lamahewa was a PhD student working in the area of medically unexplained symptoms focusing on patient needs and experiences in the Department of Primary Care and Population Health, UCL.

For peer review only

Marta Buszewicz, MRCGP, MRCPsych. is a part-time GP and Reader in Primary Care at the Department of Primary Care & Population Health, UCL.

For peer review only

References

1. The Academy of Royal Medical Colleges. The UK Foundation Programme Curriculum. July 2012. http://www.foundationprogramme.nhs.uk/download.asp?file=FP_Curriculum_2012_Updated_for_Aug_2015_-_FINAL.pdf.
2. Wessley S, Nimnuan C, Sharpe M. Functional somatic syndromes: one or many? *Lancet* 1999; 354:936-9.
3. Haller H, Cramer H, Lauche R, et al. Somatoform disorders and medically unexplained symptoms in primary care: A systematic review and meta-analysis of prevalence. *Deutsches Arzteblatt International* 2015; 112:279-287.
4. Aggarwal V, Mcbeth J, Zakrzewska J, Lunt M, Macfarlane G. The epidemiology of chronic syndromes that are frequently unexplained: do they have common associated factors? *Int J Epidemiol* 2006; 35:468-476.
5. Nimnuan C, Hotopf M, Wesseley S. Medically Unexplained Symptoms: an epidemiological study in seven specialities. *J Psychosom Res* 2001; 51:361-367.
6. Salmon P, Ring A, Dowrick D, et al. What do general practice patients want when they present medically unexplained symptoms, and why do their doctors feel pressurized? *J Psychosom Res* 2005; 59:255-260.
7. Wileman L, May C, Chew-Graham CA. Medically unexplained symptoms and the problem of power in the primary care consultation: a qualitative study. *J Fam Pract* 2002; 19:178-82.
8. Peters, S, Rogers A, Salmon P, Gask L, Dowrick C, Morriss R. What do patients choose to tell their doctors? Qualitative analysis of potential barriers to reattributing medically unexplained symptoms. *J Gen Intern Med* 2009; 69:571-7.
9. Bermingham S, Cohen A, Hague J, Parsonage M. The cost of somatisation among the working-age population in England for the year 2008–2009. *Ment Health Fam Med* 2010; 7:71-84.
10. Hatcher S, Arroll B. Assessment and management of medically unexplained symptoms. *BMJ* 2008; 336:1124-1128.
11. Kouyanou, K., Pither, C. E., & Wessely, S. Iatrogenic factors and chronic pain. *Psychosomatic Medicine* 1997 59:597–604.
12. van Dessel N, den Boeft M, van der Wouden JC, Kleinstauber M, Leone SS, Terluin B, Numans ME, van der Horst HE, van Marwijk H. Non-pharmacological

For peer review only - http://bmjopen.bmj.com/site/about/guidelines.xhtml

interventions for somatoform disorders and medically unexplained physical symptoms (MUPS) in adults (Review). The Cochrane Library 2014; 11.

13. Rosendal M, Blankenstein AH, Morriss R, Fink P, Sharpe M, Burton C. Enhanced care by generalists for functional somatic symptoms and disorders in primary care (Review). The Cochrane Library 2013; 9.
14. Shattock L, Williamson H, Caldwell K, *et al*. 'They've just got symptoms without science': Medical trainees' acquisition of negative attitudes towards patients with medically unexplained symptoms. *Patient Educ Couns* 2013; 91:249-254.
15. Howman M, Walters K, Rosenthal J, *et al.* Teaching about medically unexplained symptoms at medical schools in the United Kingdom. *Med Teach* 2012; 34:327-329.
16. Yon K, Nettleton S, Walters K, Lamahewa K, Buszewicz M. Junior Doctors' Experiences of Managing Patients with Medically Unexplained Symptoms: A Qualitative Study. *BMJ Open* 2015; 5:e009593. doi:10.1136/bmjopen-2015-009593
17. Walters P, Tylee A, Fisher J, Goldberg D. Teaching junior doctors to manage patients who somatise: is it possible in an afternoon? *Med Educ* 2007; 41:995-1001.
18. Aiarzaguena JM, Grandes G, Gaminde I, Salazar A, Sanchez A, Arino J. A randomised controlled clinical trial of a psychosocial and communication intervention carried out by GPs for patients with medically unexplained symptoms. Psychological Medicine 2006; 37:283-294.
19. Rosendal M, Olesen F, Fink P, Toft T, Sokolowski I, Bro F. A randomized controlled trial of brief training in the assessment and treatment of somatization in primary care: effects on patient outcome. General Hospital Psychiatry 2007; 29:364-373.
20. Rief W, Martin A, Rauh E, Zech T, Bender A. Evaluation of general practitioners' training: how to manage patients with unexplained physical symptoms. Psychosomatics 2006; 47:304-311.
21. Morriss R, Dowrick C, Salmon P, Peters S, Dunn G, Rogers A, Lewis B, Charles-Jones H, Hogg J, Clifford R, Rigby C, Gask L. Cluster randomised controlled trial of training practices in reattribution for medically unexplained symptoms. *Brit J Psychiat* 2007; 191:536-542.
22. Weiland A, Blankenstein AH, Van Saase JLCM, Van der Molen HT, Jacobs ME, Abels DC, Kose N, Van Dulmen S, Vernhour RM, Arends LR. Training medical specialists to communicate better with patients with medically unexplained physical symptoms (MUPS). A randomized, controlled trial. *PLoS ONE* 2015; 10:e0138342. doi:10.1371/journal.pone.0138342

For peer review only - http://bmjopen.bmj.com/site/about/guidelines.xhtml

23. Weiland A, Van de Kraats RE, Blankenstein AH, Van Saase JL, Van der Molen HT, Bramer WM, Van Dulmen AM, Arends LR. Encounters between medical specialists and patients with medically unexplained physical symptoms; influences of communication on patient outcomes and use of health care: a literature overview. *Perspectives on Medical Education* 2012; 1:192-206.
24. Salmon P. Conflict, collusion of collaboration in consultations about medically unexplained symptoms: the need for a curriculum of medical explanation. *Patient Educ Couns* 2007; 67:246–54.
25. Burton C, Lucassen P, Aamland A, Olde Hartman T. Explaining symptoms after negative tests: towards a rational explanation. *J R Soc Med* 2015; 108:84–88.
26. Berkhof M, van Rijssen JH, Schellart AJM, Anema JR, van der Beek AJ. Effective training strategies for teaching communication skills to physicians: An overview of systematic reviews. *Patient Education and Counseling* 2011; 84:152-162.
27. Shahriari M. Case based teaching at the bedside versus in the classroom for undergraduates and residents of paediatrics. *J Adv Med Educ Prof* 2014; 2:135-6.
28. Bullock A, Barnes E, Ryan B, Sheen N. Case based discussion supporting learning and practice in optometry. *Ophtalmic physiol Opt* 2014; 34:614-21.
29. Onyura B, Baker L, Cameron B, Friesen F, Leslie K. Evidence for curricular and instructional design approaches in undergraduate medical education: An umbrella review. *Med Teach* 2015; 10:1–12.
30. Kommalage M, Senadheera C. Using video to introduce clinical materials. *Clin Teach* 2012; 9:248-52.
31. Bordage G, Carlin B, Mazmanian PE. Continuing medical education effect on physician knowledge: effectiveness of continuing medical education: American College of Chest Physicians Evidence-Based Educational Guidelines. *Chest* 2009; 135:29S-36S.
32. Marinopoulos SS, Dorman T, Ratanawongsa N, Wilson LM, Ashar BH, Magaziner JL, Miller RG, Thomas PA, Prokopowicz GP, Qayyum R, Bass EB. Effectiveness of continuing medical education. *Evid Rep Technol Assess* 2007; 149:1-69.
33. Mazmanian PE, Davis DA, Galbraith R. Continuing medical education effect on clinical outcomes: effectiveness of continuing medical education: American College of Chest Physicians Evidence-Based Educational Guidelines. *Chest* 2009; 135:49S-55S.

For peer review only - http://bmjopen.bmj.com/site/about/guidelines.xhtml

34. Ravenzwaaij J van, olde Hartman TC, Ravesteijn H van, Eveleigh R, Rijswijk E van, Lucassen PLBJ. Explanatory models of medically unexplained symptoms: a qualitative analysis of the literature. *Ment Health Fam Med* 2010; 7:223-31.

For peer review only

For peer review only - http://bmjopen.bmj.com/site/about/guidelines.xhtml

Thank you for taking the time to complete this short Expert Consultation Exercise. This exercise asks for your recommendations for the development of an educational intervention for FY1/FY2 doctors working with patients with medically unexplained symptoms (MUS) and should take no more than 5-10 minutes of your time.

Please note that neither you nor your institution will be identified in any publications.

We will share anonymised findings with respondents at the conclusion of the study.

Please click 'next' to begin the survey.

1. Foundation Programme:

2. NHS Trust:

3. Your Job Title (including medical speciality):

Medically unexplained symptoms (MUS) can be defined as physical symptoms where no clear organic pathology is found. Examples include symptoms such as chest pain, fatigue, palpitations and shortness of breath; and syndromes such as IBS, fibromyalgia and chronic fatigue syndrome. Sometimes other terminology is used, e.g. functional disorders.

4. Does teaching about medically unexplained symptoms (MUS) form part of the teaching that FY1/FY2 doctors receive as part of their Foundation Training Programme?

- Yes
- No

For peer review only - http://bmjopen.bmj.com/site/about/guidelines.xhtml

5. If so, does this take the format of a formal teaching session?

- Yes
- No
- Not Applicable

6. If yes, could you give a brief description of the content of any formal teaching given on the topic of MUS, and the amount of time spent on this?

7. Is there any reference made (either within MUS teaching or elsewhere) to the topic of 'avoiding overinvestigation'?

- Yes
- No
- Not Applicable

8. If yes, please give brief details below:

9. Do you have any formal evaluation of the teaching provided on either the topic of 'MUS' or 'avoiding overinvestigation'?

- Yes
- No
- Not Applicable

10. If yes, please give brief details of the method of evaluation used:

For peer review only - http://bmjopen.bmj.com/site/about/guidelines.xhtml

11. Whether or not you provide any formal teaching about 'MUS' or 'avoiding overinvestigation', do these topics come up in any case-based discussions/Balint type groups which you may run as part of your course?

- ○ Yes
- ○ No
- ○ Not Applicable

12. If so, please could you give brief details below:

13. What would you consider to be an ideal method of teaching about the topic of MUS? (Tick all that apply)

- ☐ Lecture/seminar
- ☐ Case-based group discussions
- ☐ Role play (with simulated patient)
- ☐ Role play (with peers)
- ☐ One-to-one supervision
- ☐ Ward-based teaching
- ☐ GP/outpatient based teaching
- ☐ Advanced consultation skills training

Other (please specify)

14. What would you consider to be the most feasible method of teaching about the topic of MUS? (Tick all that apply)

- ☐ Lecture/seminar
- ☐ Case-based group discussions
- ☐ Role play (with simulated patient)
- ☐ Role play (with peers)
- ☐ One-to-one supervision
- ☐ Ward-based teaching
- ☐ GP/outpatient based teaching
- ☐ Advanced consultation skills training

Other (please specify)

15. How many hours of teaching on the topic of MUS would you recommend per year at:

FY1 Level

FY2 Level

16. What barriers do you perceive to the delivery of teaching on this topic?

17. Please use the space below if you have any other comments or recommendations for teaching on this topic:

18. Would you be willing to share any details of teaching resources or evaluation methods you may have on these topics with other teachers?

- ○ Yes
- ○ No
- ○ Not Applicable

19. And finally, would you be interested in taking part in a workshop to discuss the development of an educational intervention for FY1/FY2 doctors working with patients with medically unexplained symptoms (MUS)?

- Yes
- Maybe
- No

20. If you answered yes or maybe to Q18 or Q19 please provide your contact details in the space below:

Name

Email Address

Thank you very much for your recommendations
